# On-Line Optical Monitoring of the Mixing Performance in Co-Rotating Twin-Screw Extruders

**DOI:** 10.3390/polym14061152

**Published:** 2022-03-14

**Authors:** Felipe Bernardo, José A. Covas, Sebastião V. Canevarolo

**Affiliations:** 1 Graduate Program in Materials Science and Engineering, Federal University of São Carlos, Rod. Washington Luiz, km 235 SP-310, São Carlos 13565-905, Brazil; felipeocb@hotmail.com; 2 Institute for Polymers and Composites (IPC), University of Minho, Campus de Azurém, 4800-058 Guimarães, Portugal; 3 Department of Materials Engineering, Federal University of São Carlos, Rod. Washington Luiz, km 235 SP-310, São Carlos 13565-905, Brazil

**Keywords:** twin-screw extruders, mixing performance, on-line monitoring, optical characterization, kneading block

## Abstract

The use of real-time techniques to evaluate the global mixing performance of co-rotating twin-screw extruders is well consolidated, but much less is reported on the specific contribution of individual screw zones. This work uses on-line flow turbidity and birefringence to ascertain the mixing performance of kneading blocks with different geometries. For this purpose, one of the barrel segments of the extruder was modified in order to incorporate four sampling devices and slit dies containing optical windows were attached to them. The experiments consisted in reaching steady extrusion and then adding a small amount of tracer. Upon opening each sampling device, material was laterally detoured from the local screw channel, and its turbidity and birefringence were measured by the optical detector. Residence time distribution curves (RTD) were obtained at various axial positions along three different kneading blocks and under a range of screw speeds. It is hypothesized that K, a parameter related to the area under each RTD curve, is a good indicator of dispersive mixing, whereas variance can be used to assess distributive mixing. The experimental data confirmed that these mixing indices are sensitive to changes in processing conditions, and that they translate the expected behavior of each kneading block geometry.

## 1. Introduction

Mixing in an essential feature of any polymer processing routine—but particularly significant in compounding operations—when additivation, polymer blending, or polymer reinforcing are carried out. Generally, it involves the spatial re-arrangement of the formulation components, leading to uniformity by imposing a certain shear deformation history (distributive mixing), as well as the progressive decrease in size of initial agglomerates, or droplets of a suspended liquid phase, by the exertion of hydrodynamic stresses during a certain period (dispersive mixing) [1]. The complete description of the state of mixing of a given system requires the identification of the size, shape, orientation, and spatial location of every particle or droplet of the minor component along the processing equipment [2,3]. This must be obtained either through numerical modeling or experimentally, which is not straightforward, making practical mixing assessment during processing a complex topic.

Mixing with co-rotating twin-screw extruders (TSE) has been the focus of numerous modeling and experimental studies, which usually aim to characterize either the distributive or the dispersive aspects of the process. In the case of distributive mixing, Fard et al. [4] proposed a mapping method based on the tracking of particles in the velocity fields, while Wang et al. [5] calculated the evolution of the Renyi relative entropies of the minor component along the extruder. However, most studies predicted and/or measured the residence time distribution (RTD) [6,7,8,9,10]. The width of the RTD curves is generally considered a measure of distributive mixing, but other parameters of the curves (e.g., minimum and mean residence times) can be used to gain an understanding of the effect of screw geometry and operating conditions on the machine’s response. Various approaches to model and/or measure experimental RTD curves in TSE have been reported [11,12,13,14,15,16,17,18]. The assessment of dispersive mixing in TSE has deserved less attention in the literature, as it requires a precise (3D numerical) description of the flow together with information on local residence times and hydrodynamic stresses developing along the extruder. For a liquid–liquid system, Emin and Schuchmann [19] implemented the calculation of the critical capillary number into particle tracking simulations. For a suspension of solids in a liquid, Manas-Zloczower and co-authors [1,20] put forward an agglomerate dispersion model considering rupture and erosion routes that will develop depending on the magnitude of a local fragmentation number, which is also associated with a finite probability to break-up. Valette et al. [21] applied a dispersion kinetic theory to predict mass density distributions.

A few authors have also proposed the use of mixing indices to straightforwardly characterize the extent of distributive or dispersive mixing, as these would facilitate direct comparisons between different operating conditions, screw profiles, and material properties. Average particle/droplet size relative to the initial size, number-average diameter [22,23], relative flow strength [24], shear stress distribution [25,26], and cumulative area ratio [27,28] have been routinely used as indicators of dispersive mixing, whereas Shannon entropy [29] was suggested as a measure of distributive mixing. Nevertheless, these indices require either lengthy experimental characterization or computationally demanding calculations. Teixeira et al. [30] evaluated the performance of a few mixing indices and concluded that the variance presents high statistical variability and seems to be the most adequate for flow regimes where the mixing level is low. 

In-process monitoring techniques (i.e., techniques that allow measurements to be performed directly in the extruder during its operation) have been gradually applied to TSE, as they offer the obvious advantages of directly probing the material while it is being processed, minimizing the delay between sample collection and measurement, reducing or avoiding sample preparation, facilitating measurements at regular time intervals, and thus supporting quality and process control schemes. On-line measurements are performed on a portion of material detoured from the main flow, whereas in-line measurements are made directly on the latter (an excellent review of in-process techniques applied to hot melt extrusion is available [31]). Optical techniques are quite attractive for in-process monitoring, since they have fast response times and do not disturb the environment being analyzed. Small-angle light scattering (SALS) yields spatial information on flow-induced structures [32], whereas light beam attenuation, measured as birefringence or turbidity, is related to orientation [33] or dispersion of a second phase [34], providing information on shape [35] or size [36,37] of dispersed particles. 

Recently, Bicalho et al. [38] performed turbidity and birefringence measurements along a TSE to monitor the melting of polypropylene. By inserting a tracer pulse at the entrance of the TSE and measuring its concentration with time in terms of turbidity at selected locations downstream, RTD curves were obtained. The latter were sensitive to axial location, barrel temperature, and screw speed. 

Building on this development, this work aims at using information extracted from RTD curves obtained by means of on-line turbidity and birefringence measurements to characterize the distributive and dispersive mixing abilities of kneading blocks with different geometries of a TSE, operating under a range of screw speeds. For this purpose, a blend of PS/PA6 is processed, PS being the matrix and PA6 the tracer. PS is an amorphous polar polymer, often adopted for melt mixing studies, presenting an easily measurable flow birefringence, one of the optical parameters of interest. PA6 is a semi-crystalline polymer, with a well-defined melting temperature (T_m_~225 °C), that is thermodynamically immiscible with the PS matrix, which is a fundamental requirement for turbidity measurements. However, in principle, any other immiscible polymer blend would be adequate for this study. Measurements are made at various axial locations, thus evidencing the kinetics of mixing along each kneading block. The real-time data obtained confirmed that this novel approach is able to adequately identify the expected effect of processing conditions and kneading block geometry on distributive and dispersive mixing. 

The paper is organized as follows. The optical measurements and the assessment of mixing with RTD curves are first discussed. The experimental set-up and procedure are explained. Data related to axial pressure, melt temperature, specific mechanical energy (SME), and RTD are presented and used to describe flow in the mixing zone. Then, the relevant parameters and mixing indices extracted from the RTD curves are used to characterize mixing along the kneading blocks with different geometries.

## 2. Theoretical Background

### 2.1. Light Interaction with Matter

When light travels through matter, four phenomena may result from this interaction [39,40]. Part of the radiation may be: (a) reflected at the interface (*I_R_*); (b) refracted, i.e., transmitted through the material with a change in its propagation direction (*I_T_*); (c) transmitted in the same propagation direction (*I*); and (d) absorbed (*I_A_*). The reflected and refracted components are taken as light scattering, which is characterized by changes in the radiation propagation direction. Then, the attenuation of the light beam with incident light intensity *I*_0_ while interacting with matter, is given by

(1) $$I_{0}-I=I_{R}+I_{T}+I_{A}$$

where *I_R_* and *I_A_* are the reflected and absorbed light beam intensities, respectively, and *I_T_* and *I* are the transmitted light beam intensity with and without change in propagation direction, respectively.

Turbidity (τ) can be defined as the attenuation of a transmitted light beam when passing through a medium. In polymers, turbidity is dependent upon the type, volume fraction, particle size, and shape of the disperse phase, i.e., it increases with increasing number of particles [41]. In a population of particles, it also increases when the concentration of particles whose sizes are close to the visible light wavelength range increases [42]. Turbidity can be used to analyze the dispersion of solid particles in polymers, because they act as scattering obstacles to the passage of light. Particles can also be taken as tracers, for example, revealing their position in a flowing medium [37]. Due to the appearance of multiple scattering effects at large numbers of particles, the detection is limited to low volume concentrations of the disperse phase, typically up to 5% w/w, under which the linearity of the detector signal is obtained. The transmitted light intensity measured by an optical detector can be normalized ($V_{N}$) to a dimensionless value between zero and one [43]

(2) $$V_{N}=\frac{V-V_{0}}{V_{s}-V_{0}}$$

where V is the measured sample response voltage, $V_{s}$ is the saturation detector response measured by switching off the light source, and $V_{0}$ is the response voltage of the flow. These two control voltages are measured periodically and whenever the optical detector changes location.

Birefringence (∆n) can be defined as an optical property of anisotropic materials and it is, by definition, the maximum difference between two refractive indexes orthogonally oriented with respect to each other [44]. Birefringence is commonly used to estimate the level of orientation of polymer chains with the flow. In a quiescent melt there is no orientation, therefore the polymer chains are in a random conformation and are optically isotropic. However, during flow, the velocity gradient induces orientation and thus generates optical anisotropy, creating flow birefringence. To obtain the birefringence of a polymeric sample, the relative retardation (δ) between the polarized light rays that cross it is determined. The cross-polarized transmitted light intensity ($I^{B}$) measured by an optical detector can be normalized to a dimensionless value ($I^{B}{}_{N}$) set between zero and one, using the minimum intensity while the polarizers are crossed ($I_{C}$) and the maximum intensity while parallel ($I_{p}$), as defined by

(3) $$I^{B}{}_{N}=\frac{I^{B}-I_{C}}{I_{P}-I_{C}}\;.$$

$I^{B}{}_{N}$ is related to the optical path difference (OPD) of an anisotropic material placed between crossed polarizers and oriented at 45° with respect to their optical axes by a simplified version of Malus’ law [44]

(4) $$I^{B}{}_{N}=\sin^{2}\frac{\delta }{2}=\sin^{2}\left(\frac{\pi \;OPD}{\lambda }\right)\;$$

where λ is the wavelength of the light source (taken as 550 nm for visible light) and δ is the relative retardation of the polarized light ray.

The normalized cross-polarized transmitted light intensity ($I^{B}{}_{N})$ of a liquid–liquid suspension results from all optical effects, which include birefringence and turbidity. In turn, the total birefringence induced by the velocity gradient during flow encompasses flow birefringence (Δ$n_{S}$) due to chain orientation of the polymer matrix, and a form birefringence (Δ$n_{F}$) due to the deformation of the dispersed phase. Initially, the latter is approximately spherical but changes into a rod-like geometry with flow initiation. For high concentrations of the second phase, a lamellar morphology may also be formed. Form birefringence is a function of the refractive indices, volumetric fractions, and phase morphology [45,46]; it is zero for undeformed dispersed spheres, positive for dispersed rods, and negative for lamellar morphologies. Then, the total measured normalized cross-polarized transmitted light intensity is the summation of all optical effects, including the contribution of flow (Δ$n_{S}$) and form (Δ$n_{F}$) birefringence, and of the turbidity (τ), with their respective signs

(5) $$I_{cross\;polarized}^{total\;measured}=\left(\pm I_{\Delta n_{S}}\right)+\left(\pm I_{\Delta n_{F}}\right)+\left(+I_{\tau }\right).$$

This simple relation holds, and can be applied when the total optical path difference (OPD) is within the first half of the first order, i.e., OPD < 250 nm, in the cross-polarized light interference chart. Knowing that the dispersed phase morphology changes from spheres to rods, the form birefringence can be estimated from the normalized cross-polarized transmitted light intensity

(6) $$I_{\Delta n_{F}}=I_{cross\;polarized}^{total\;measured}-I_{\tau }$$

### 2.2. Assessment of the Mixing Level with RTD Curves

The concept of residence time distribution (RTD) was introduced in 1953 by Danckwerts as a means to describe non-ideal liquid mixing in chemical reactors [47]. Thenceforth, RTD curves have been extensively used to examine the hydrodynamics and mixing behavior in many processes. This technique, which in the case of blending systems aims at describing the macro-mixing dynamics (i.e., the movement of particles inside the system along a major axis), is performed by introducing a traceable material (hereon referred to as ‘tracer’) at a known position and then tracking its concentration as it exits the system [48]. RTD represents the output tracer concentration (normalized by the area under the curve) versus time, as *E*(*t*):

(7) $$E\left(t\right)=\frac{c\left(t\right)}{\int_{0}^{\infty }c\left(t\right)dt}.$$

Since differences in the distribution and dispersion of a polymeric second phase suspended in the main flow affect the optical behavior of the flowing melt, the measurement of RTD curves using optical detection can be used to estimate the mixing performance during extrusion. RTD curves can be obtained by measuring synchronously melt flow turbidity and birefringence. The application of both optical properties is particularly interesting compared to other more conventional measurements, because they are a function of the number, size (equivalent diameter), and shape of the scattering particles present in the flow, which is not the case for RTDs obtained from the concentration distribution of a tracer.

The mean (*t_n_*) residence time is an obvious RTD parameter that can be used to evaluate mixing. It can be calculated by [18]

(8) $$t_{n}=\frac{\int_{0}^{\infty }ctdt}{\int_{0}^{\infty }cdt}=\frac{\sum_{0}^{\infty }ct\Delta t}{\sum_{0}^{\infty }c\Delta t}=\frac{\sum_{t_{i}}^{t_{f}}V_{Ni}*t_{i}*\left(t_{i}-t_{i-1}\right)}{\sum_{t_{i}}^{t_{f}}V_{Ni}*\left(t_{i}-t_{i-1}\right)}$$

where *c* is the tracer/pulse concentration at time t, which is taken here as $V_{N}$, the normalized transmitted light intensity response, calculated by Equation (2); *t_i_* is the minimum residence time, i.e., the time required for the first particles of the suspended phase to be detected; *t_f_* the maximum residence time, i.e., the time elapsed until the suspended phase is no longer detected; ∆*t* the time interval determined by the on-line data collection frequency, usually at 5 Hz or 0.2 s.

The total area under a RTD curve (*A*) is a direct and quantitative measure of tracer content, and can be calculated from

(9) $$A=\int_{0}^{\infty }cdt=\overset{t_{f}}{\underset{t_{i}}{\sum }}c_{i}*\Delta t=\overset{t_{f}}{\underset{t_{i}}{\sum }}V_{Ni}*\left(t_{i}-t_{i-1}\right).\;$$

The concentration *c* can be measured optically following the melt flow turbidity (which is the reduction in transmitted light intensity), which depends on the number and size of the scattering particles. The more the second phase is dispersed in the matrix, the higher the number and the smaller the particles, increasing turbidity. Therefore, *A* can be used to estimate the extent of dispersive mixing (its value increasing with mixing intensity). On the other hand, the variance ($\sigma^{2}$) of the RTD curve

(10) $$\sigma^{2}=\int_{0}^{\infty }{\left(t-t_{n}\right)}^{2}E\left(t\right)dt=\overset{\infty }{\underset{0}{\sum }}{\left(t-t_{n}\right)}^{2}E\left(t\right)\Delta t\;$$

correlates with the distributive mixing character of the process, as its value quantifies the spreading of the concentration with time. In this equation, *E*(*t*) is the residence distribution function, and $t_{n}$ is the mean residence time.

To better analyze the experimental RTD curves obtained from the measurements, they were fitted by theoretical pulse curves [49]

(11) $$I_{t\;}=I_{0\;}+K\;{\left[1-e^{-\left(\frac{t-t_{i}}{R_{1}}\right)}\right]}^{p}\times e^{-\left(\frac{t-t_{i}}{R_{2}}\right)}$$

where *I*_0_ is the initial intensity or base line value (here set to zero), K is an area constant, *t_i_* is the minimum residence time, *R*_1_ and *R*_2_ are the rise and fall time rates, respectively, and p is a power exponent. *R*_1_ relates to the first part of the RTD curve, before the maximum, hence representing its rise time rate. The higher the *R*_1_, the lower this rate. *R*_2_ is associated with the region of the RTD curve beyond the maximum, thus quantifying its fall time rate. Again, the greater its value, the lower the rate of the RTD curve returning to its base line. The p parameter shifts the peak of the RTD curve down and forward, thus spreading the curve and reducing its area. This means a reduction in the number of particles and an increase in the axial spreading of the dispersed phase. Changes in p are much more perceptive than changes in *R*_2_. *R*_1_ is even less sensitive, as it is obtained from fitting a short portion of curve. K is a constant related to the area under the curve, and is very sensitive both to changes in intensity of the RTD, and to changes in *R*_1_, *R*_2_, and p, i.e., with changes in the number and dispersion of particles.

Here, we propose using the parameter K as an indicator of dispersive mixing performance, whereas the variance is used to assess distributive mixing. Both were chosen because they are related to the number and shape of dispersed particles, and they are simple and easy to quantify, especially when compared to other more widespread mixing indicators.

## 3. Materials and Methods

### 3.1. Materials

A commercial grade of a general-purpose polystyrene, PS (Styrolution 124 N/L, manufactured by INEOS Styrolution, Frankfurt, Germany), with MVR of 12 cm^3^/10 min (5.0 kg, 200 °C) and density of 1.04 g/cm^3^ was extruded as matrix. A polyamide 6, PA6 (Domamid^®^ 6NC01, manufactured by DOMO Chemicals, Leuna, Germany), with MVR of 165 cm^3^/10 min (5.0 kg, 275 °C) and density of 1.00 g/cm^3^ was used as tracer/pulse.

### 3.2. Experimental Set-Up

**Figure 1:**
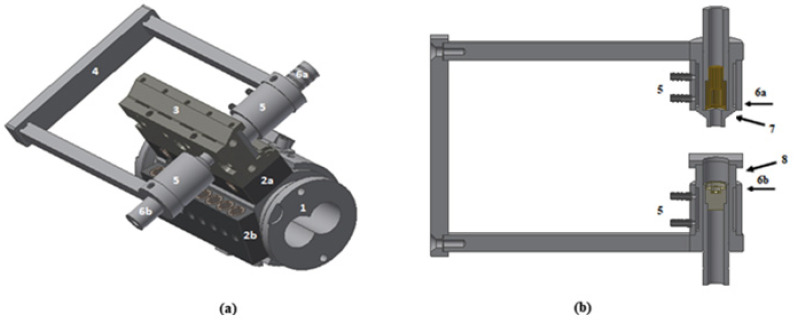
(**a**) Experimental set-up: modified barrel segment (1) with two axial rows of sampling devices (2a, 2b); multi-slit die (3); sliding optical detector with a C-shaped support (4); water-cooling system (5); light source (6a) and receptor (6b) for optical measurements and (**b**) Optical detector system: LED (6a) with a polarizer (7) and two LDRs (6b) with a polarizing filter (8).

The experiments were performed in a modular co-rotating intermeshing twin-screw extruder Collin ZK 25P (COLLIN Lab & Pilot Solutions, Maitenbeth, Germany) with a screw diameter of 25 mm and L/D = 48. The modular barrel is composed of eight interchangeable segments, each with its own temperature control. As illustrated in Figure 1, one of these was replaced by a modified segment (1) containing two axial rows of sampling devices (2a and 2b). Each device consists of an on–off valve which, when rotated 90°, allows the material to flow out of the extruder, along a circular side-channel linking the inner and outer barrel walls. A multi-slit die (3) (containing 4 slits, each 30 mm long, 15 mm wide, and 1.5 mm thick, each aligned with the circular channel of one sampling device through a conical connection) was fixed to the top row of the sampling devices (2a). Each slit contains a pair of directly opposed transparent circular windows with a diameter of 10 mm, so that changes in light intensity transmitted through the polymer melt flow can be analyzed by an optical detection system (4–6). The latter contains an aligned pair of light emitters (6a) and light receiver (6b), which are kept in position by a C-shaped support (4). The entire contrivance can slide axially along the barrel segment, in order to make measurements at the various slit dies. A white LED (light emitting diode) with a polarizer (7) was used as light source and two LDRs (light dependent resistor) as photodetectors, the first one to quantify changes in the light beam intensity, i.e., in melt flow turbidity; and the second one, positioned behind a polarizing filter (8) and aligned 45° with respect to the flow and 90° to the LED’s polarizer, to quantify birefringence. The signals from the two LDRs synchronously measuring turbidity and birefringence are: (i) collected at a frequency of 0.1 MHz (with an accuracy of 5%), (ii) converted into digital signals by means of an analogic-digital interface (USB data acquisition NI-DAQ 6812), (iii) transmitted to a personal computer running the software developed in the LabVIEW 8.6 NI platform (National Instruments) which averages (compresses) the data to present it at 10 Hz, (iv) makes the real-time calculations, (v) screen presentation, and (vi) data saving. Data collection should not be affected by the inherent extruder vibrations caused by the drive elements, as the corresponding frequency ranges are quite distinct (0.1 MHz for data collection, 0–100 Hz for the mechanical vibrations [50]). A detailed description of the set-up can also be found elsewhere [38].

**Figure 2:**
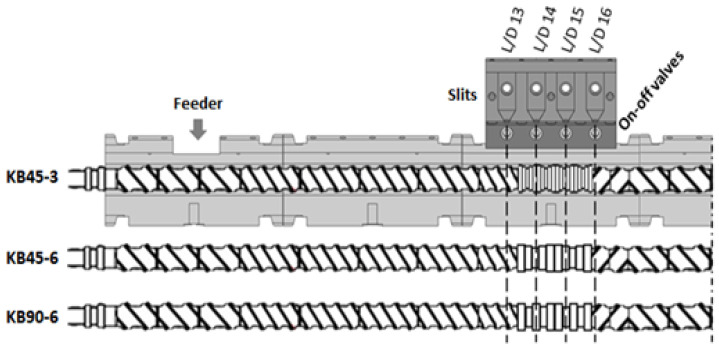
Screw profiles containing a 60 mm long mixing zone with different geometries: KB45-3—four kneading blocks with positive 45° stagger, each containing five 3 mm thick disks; KB45-6—two kneading blocks with 45° positive stagger, each containing five 6 mm thick disks; KB90-6—two kneading blocks with neutral 90° stagger, each containing five 6 mm thick disks.

Figure 2 presents the upstream part of the extruder, including the modified barrel segment, together with the three screw profiles studied. The geometry downstream was kept constant in all experiments. The feed rate is controlled by a K-Tron gravimetric feeder. The collecting ports are located at L/D = 13, 14, 15, and 16. Since the aim here is to investigate the influence of screw design on the mixing efficiency:

(i) The three screws have the same configuration up to L/D = 13, consisting of conveying elements with decreasing pitch downstream.
(ii) The three screws have the same configuration from L/D = 16 onwards, starting with two left-handed (LH) elements (each 15 mm long), in order to ensure that they worked fully filled upstream, at least at L/D = 16, so that a material sample could be collected for an optical measurement.
(iii) Between L/D = 13 and L/D = 16, three distinct mixing zones were assembled: (1) four kneading blocks with positive 45° stagger, each containing five 3 mm thick disks (KB45-3); (2) two kneading blocks with 45° positive stagger, each containing five 6 mm thick disks (KB45-6); (3) two kneading blocks with neutral 90° stagger, each containing five 6 mm thick disks (KB90-6).

### 3.3. Experimental Procedure

In order to keep the experimental effort within reasonable limits, all the experiments were performed with a feed rate of 2 ± 0.1 kg/h and a uniform barrel temperature set to 230 ± 1 °C, while the screw speed was varied between 50 rpm and 500 rpm, in order to generate different hydrodynamic stress levels and degrees of channel fill—and, consequently, different melting rates and degrees of mixing—while avoiding excessive shear rates inducing polymer degradation.

For each processing run, upon reaching steady state extrusion of PS, a pulse of PA6 is added (0.105 g, corresponding to a concentration lower than 0.1% w/w relative to the matrix), the valve of a specific sampling device is opened and the optical detector starts synchronously recording the transmitted light intensity as turbidity and the cross-polarized transmitted light intensity as birefringence. The presence of the dispersed phase in the flow through the slit-die produces light scattering and retardation, which are recorded in real-time. In both cases, the data comes out as a typical residence time distribution (RTD) curve. 

This procedure was repeated for the three remaining sampling positions, and then the entire experiment was replicated for different screw speeds and for the various screw profiles. At least 120 RTD curves were acquired, measurements of each curve being repeated three times. When the screw channels worked partially filled locally, the melt did not flow continuously towards the corresponding slit and no measurement could be made. 

This sampling procedure may perturb the flow characteristics in the screws, both in the vicinity and downstream of the location being analyzed. However, not only should the comparison between the results of the various experiments hold, but the advantages of the method should compensate for its limitations, as it yields data at small axial increments without the need to stop the extruder (an operation that may also affect the quality of the data), which are seldom reported in the literature.

## 4. Results and Discussion

### 4.1. Flow along the Mixing Zone

Upon operation of the extruder, when opening the on–off valve of any of the sampling ports, flow of material from inside the extruder will take place if the screws work fully filled in that location. If there is no flow, then the screws are only partially filled. Moreover, the flow rate of material through any sampling port is directly proportional to the local melt pressure in the flow channel. Therefore, by measuring the flow rate out of the sampling devices at the four locations, information equivalent (and proportional) to the corresponding axial pressure development is obtained, as well as on the axial location upstream of the LH elements where the screws begin to work fully filled. 

**Figure 3:**
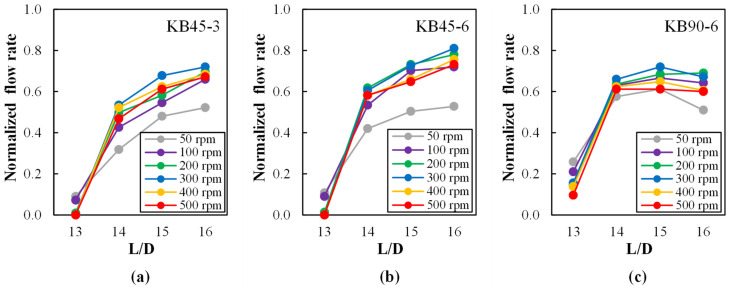
Normalized flow rate at ports L/D = 13, 14, 15, and 16 along the mixing zone as a function of screw speed (50 to 500 rpm) for each kneading block: (**a**) KB45-3, (**b**) KB45-6 and (**c**) KB90-6. The normalized flow rate is a direct indication of the local pressure inside the screw channel.

Figure 3 presents the flow rate (normalized to the extruder output) at each port (L/D = 13, 14, 15, and 16) for the three screw profiles and a range of screw speeds between 50 and 500 rpm. As depicted in Figure 2, upstream of this zone the screws are made of conveying elements, while immediately downstream two left-handed elements exist. Therefore, a progressive increase in flow rate (i.e., pressure) along this section is anticipated in order to overcome the flow resistance created downstream by the restrictive elements [51]. Additionally, both the slope of the flow rate and the length of the screws working fully filled may vary with screw geometry and screw speed. The plots in Figure 3 confirm these hypotheses, as the flow rate (i.e., pressure) increases downstream for all screws, but the initial value and the slope are different, especially for the screw containing kneading disks staggered 90° (KB90-6). This is related to the extent of channel fill. Figure 3 shows that at L/D = 13 the local flow rate for screws KB45-3 and KB45-6 is either low or nil, depending on screw speed, while the values are higher for KB90-6. Thus, one may infer that the number of fully filled conveying channels is higher for the latter, leading to a flatter axial profile. Up to 300 rpm, the maximum local flow rate (i.e., pressure) increases with increasing screw speed (the highest values being attained by the KB45-6 screw which contains thicker disks), as the higher the screw speed, the higher the flow resistance created by the left-handed elements downstream should be. Furthermore, the slope of the local flow rate increases as the number of fully filled channels upstream reduces, which can be presumed by the progressively lower, or even nil, feed rates at L/D = 13. This behavior is reversed for 400 rpm and above, as the balance between the conveying capacity of the extruder and the feed rate of 2 kg/h is altered.

**Figure 4:**
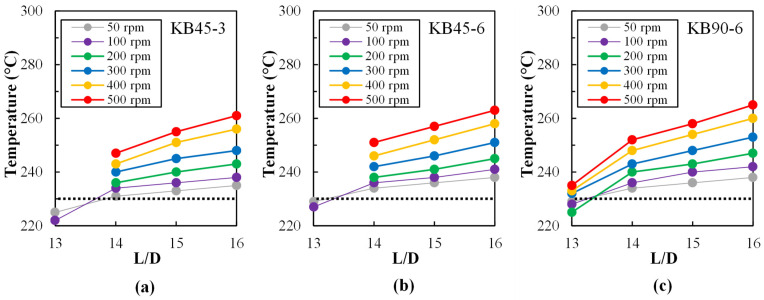
Melt flow temperature (°C) along the mixing zone for various screw speeds (50 to 500 rpm) for each kneading block: (**a**) KB45-3, (**b**) KB45-6, and (**c**) KB90-6. The set extrusion temperature (230 °C) is represented by a horizontal dashed line in each chart.

The temperature of the melt exiting each sampling device can be readily measured by sticking a fast response digital skewer thermometer into the flow, the value obtained being a good estimate of the average temperature of the material in the extruder in the vicinity of the sampling port. The data presented in Figure 4 reveals the effect of screw geometry and screw speed on the axial temperature profile between L/D = 13 and L/D = 16, the zone of the screws being studied. The values attained seem to indicate that the material was mostly molten in this region. When the screws worked partially filled at L/D = 13, it was not possible to collect this type of data (see also Figure 3). In all cases, the temperature grows as flow progresses along the mixing zone, and it increases steadily with increasing screw speed. At L/D = 16, viscous dissipation caused an overheating ranging from approximately 5 °C when the screws rotate at 50 rpm to more than 30 °C at 500 rpm. The maximum temperature probed was attained by screw KB90-6, followed by screw KB45-6 and then screw KB45-3. This result is the combined effect of hydrodynamic stress levels and residence time in each mixing zone and follows the expected behavior [52]. 

The specific mechanical energy (SME) characterizes the amount of energy per mass unit of extrudate that is transferred to the material by mechanical input during extrusion. Several authors have found correlations between the value of this parameter and certain characteristics of the extrudates, for example for polymer–clay nanocomposites [53] and in extrusion cooking [54]. When processing a polymer system that does not undergo chemical reactions and/or significant morphology changes, SME is expected to increase with increasing screw speed, and decreasing temperature or throughput [55]. It can be calculated from [56]:

(12) $$\mathrm{SME}=\frac{2\pi \omega \mathrm{To}}{\dot{m}}\times 3.6$$

where ω is the screw speed (rpm), To is the corrected torque (N.m), i.e., the ratio of the real torque during operation and the permissible torque, and $\dot{m}\;$is the feed rate (kg/h).

**Figure 5:**
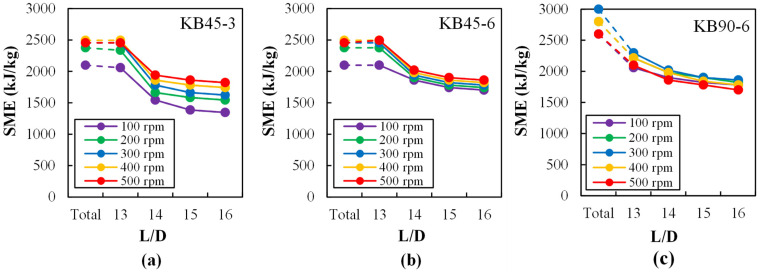
Specific mechanical energy SME (kJ/kg) at ports L/D = 13, 14, 15, and 16 along the mixing zone as a function of screw speed (50 to 500 rpm) for each kneading block: (**a**) KB45-3, (**b**) KB45-6, and (**c**) KB90-6. “Total” denotes SME values without any material sampling.

Figure 5 exhibits correlations between SME and screw speed for the various screw profiles and axial positions under investigation. Based on previous studies, it is assumed that the mechanical energy is mainly transferred in the zones containing kneading elements, whereas conveying elements require insignificant contribution [53]. The values denoted as “total” were obtained when running the extruder conventionally, without any sampling. The remaining data are rarely revealed in the literature, as it corresponds to the values of SME computed when the valve at each corresponding location is open and, in most cases as discussed above, lateral flow develops. Therefore, the magnitude of the SME decreases, since the total flow in the extruder from the sampling point onwards also reduces. The data are interesting, since the differences of SME values between two consecutive L/D locations show the contribution to the total SME. Not surprisingly, the shapes of these curves tend to mirror those of the normalized flow rates at the various ports (Figure 3), as the local mechanical energy input was largely dissipated as an increase in pressure. The trend of increasing SME with increasing screw speed attenuates at the higher screw speed range due to the joint effect of lower degree of screw fill (demonstrated in Figure 3), the pseudoplastic nature of the melt, and the viscous dissipation (displayed in Figure 4). As for the actual SME values, they tend to follow the rank KB90-6 > KB45-6 > KB45-3, in accordance with their relative restrictive character.

**Figure 6:**
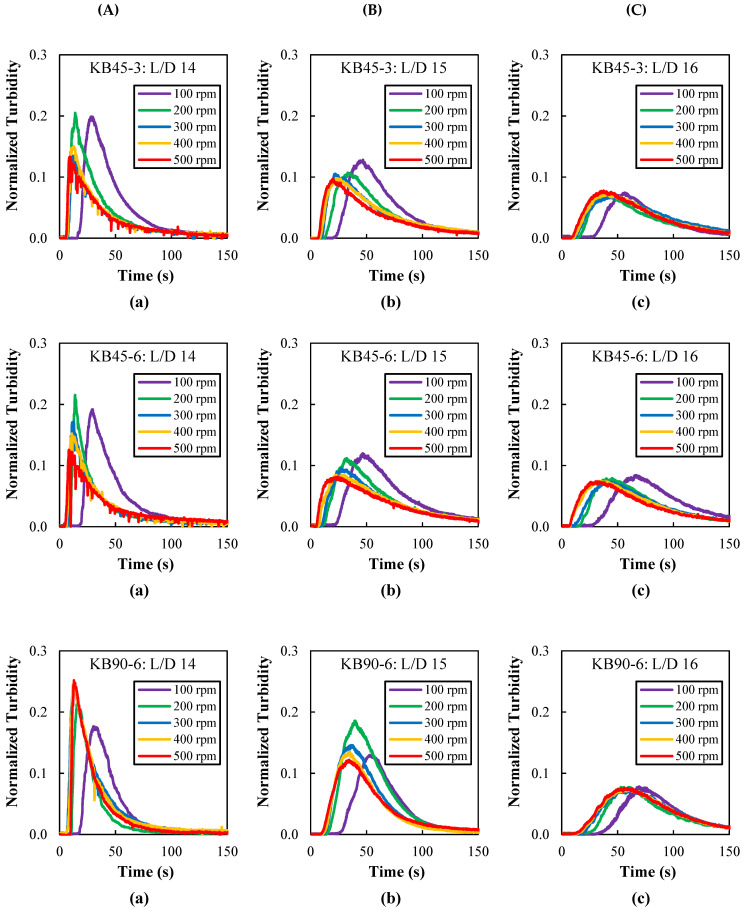
Residence time distribution (RTD) curves measured with the barrel set to 230 °C along the mixing zone at ports: (**a**) L/D = 14, (**b**) L/D = 15, and (**c**) L/D = 16 for different kneading blocks (**A**: KB45-3, **B**: KB45-6, and **C**: KB90-6) as a function of screw speed (100 to 500 rpm). The time scale was shortened to 150 s to better show the major peak area.

The RTD curves obtained by following melt flow turbidity were measured between L/D = 14 and 16, for the three screw profiles and screw speeds ranging from 100 to 500 rpm, with the barrel set to 230 °C. These curves are shown in Figure 6. Curves were also obtained for two other barrel set temperatures (220 °C and 240 °C, not shown), revealing the same pattern. All the curves exhibit the typical pulse shape, becoming broader and being shifted to longer times as they are obtained more downstream. Differently, increasing screw speeds shifts the curves to shorter times while widening them. These results were to be expected, as they reflect the typical progression of the material along the screws of a co-rotating twin screw extruder with a corresponding enhancement of mixing [51,57].

**Figure 7:**
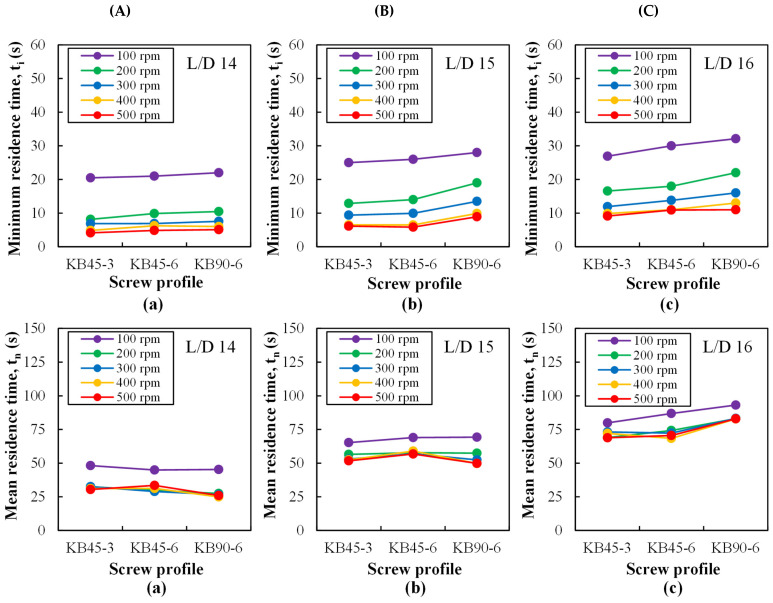
Minimum (*t_i_*) and mean (*t_n_*) residence times (s) measured with the barrel set to 230 °C along the mixing zone at ports: (**a**) L/D = 14, (**b**) L/D = 15, and (**c**) L/D = 16 for different kneading blocks (**A**: KB45-3, **B**: KB45-6, and **C**: KB90-6) and screw speeds (100 to 500 rpm).

Figure 7 presents the minimum and mean residence times extracted from the RTD curves of Figure 6. Predictably, as in [58], both increase along the screw axis and decrease with increasing screw speed, although this effect attenuates as the rotation frequency increases. Since the conveying capacity of the mixing blocks follows the rank KB45-3 > KB45-6 > KB90-6 (which has no conveying capacity), the values of the minimum time logically evidence the opposite trend. This is also true for the mean residence time at L/D = 16, as differences are more difficult to perceive at the two ports upstream.

### 4.2. Assessing Mixing

**Figure 8:**
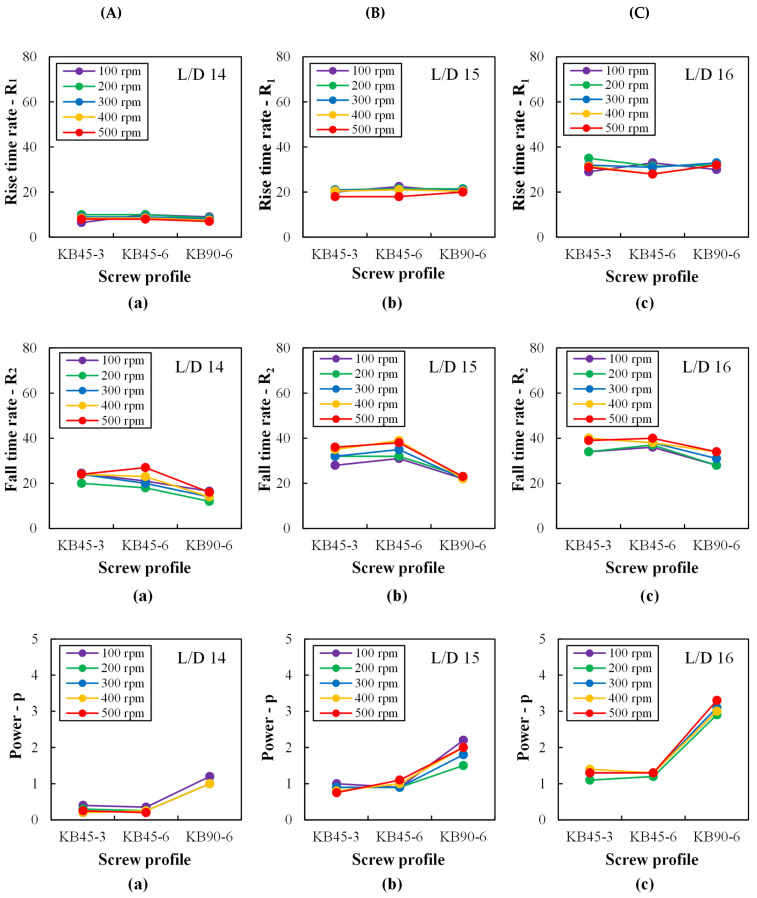
Rise (*R*_1_) and fall (*R*_2_) time rates and power exponent (p) parameters of the fitted pulse curve to the RTD curves measured with the barrel set to 230 °C along the mixing zone at ports: (**a**) L/D = 14, (**b**) L/D = 15, and (**c**) L/D = 16 for different kneading blocks (**A**: KB45-3, **B**: KB45-6, and **C**: KB90-6) and various screw speeds (100 to 500 rpm).

Having characterized the flow along the different kneading blocks of the three screws, the RTD data were used to assess mixing. Figure 8 shows the rise and fall time rates (*R*_1_ and *R*_2_ respectively), as well as the power p parameters, obtained by fitting the best pulse curve to each experimental RTD curve, as discussed above. Probably due to the difficulty in fitting a theoretical curve to the very narrow first portion of the RTD curve, R_1_ is nearly constant regardless of kneading block type or screw speed, but increases axially downstream. This increase is consistent and makes sense, since the particles’ spatial distribution should improve as the material progresses along the extruder. Within the error introduced by the fitting routine, R_2_ shows some sensitivity to changes in kneading block geometry, screw speed, and axial position. As expected, it increases as the material progresses downstream, and seems to have a tendency to increase with increasing screw speed, but the lowest values obtained for KB90-6 are unforeseen and cannot be readily explained in terms of mixing behavior. Contrariwise, the power parameter p increases steadily downstream, attains higher values for KB90-6 than for the positive stagger angles (KB45-3 and KB45-6) due to the repeated melt split and joining in this element, but shows limited sensitivity to screw speed (although with the global expected trend, i.e., an increase with increasing screw speed, especially at L/D = 15 and L/D = 16).

**Figure 9:**
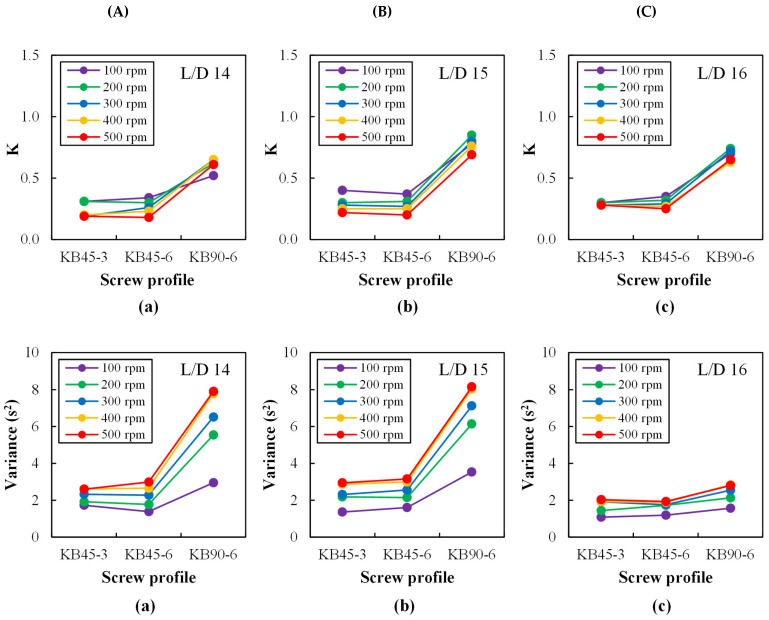
Area constant (K) and variance ($\sigma^{2}$) based on RTD curves measured with the barrel set to 230 °C along the mixing zone at ports: (**a**) L/D = 14, (**b**) L/D = 15, and (**c**) L/D = 16 for different kneading blocks (**A**: KB45-3, **B**: KB45-6, and **C**: KB90-6) and various screw speeds (100 to 500 rpm).

Figure 9 assesses the extent of dispersive and distributive mixing in terms of the area constant (K) and variance ($\sigma^{2}$), respectively, i.e., using the mixing indices suggested here (see the theoretical background section). Remarkably, the data are sensitive to changes in screw geometry, screw speed, and axial position. Dispersion progresses slightly along each of the kneading blocks, decreases with increasing screw speed, and depends on the screw configuration. It requires not only sufficiently high hydrodynamic stresses (which increase with increasing screw speed), but also that these are exerted during enough time (the mean residence time decreasing with increasing screw speed, as seen in Figure 7). Therefore, the results indicate that for the material, geometry of the kneading blocks, and operating conditions used in the experiments, residence time is the predominant effect. Similar results have been recently reported for polymer/clay nanocomposites [59]. Therefore, and as anticipated, KB90-6 is the most efficient configuration for dispersion, as it is associated to higher residence times. A distinction between KB45-3 and KB45-6 is more difficult to make, although the second performs marginally better given the use of thicker kneading disks, with less conveying capacity but more efficient smearing of the material [51]. This is probably because of the existence of the two left-handed elements immediately downstream of the mixing block, which were utilized with the aim of creating pressure upstream and thus facilitating material sampling, but probably masked somewhat the inherent role of each geometry on melting/dispersing efficiency.

**Figure 10:**
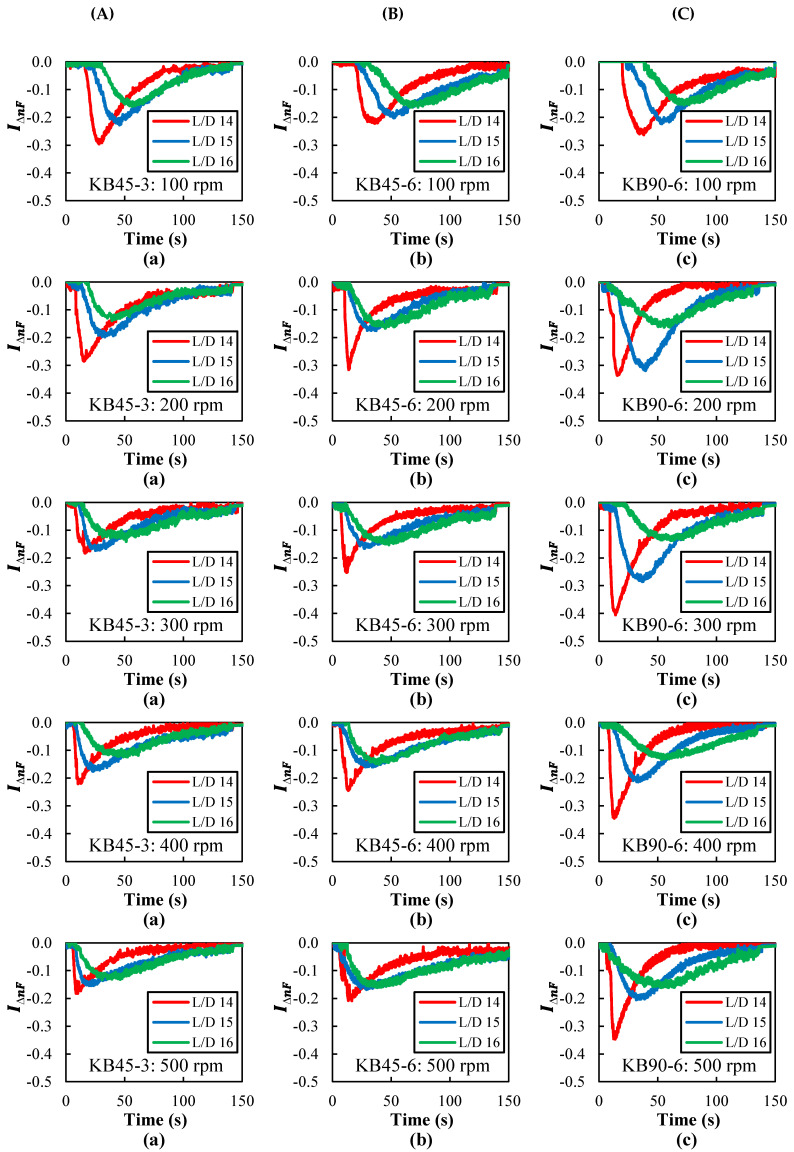
Normalized cross-polarized transmitted light intensity ($I_{\Delta n_{F}}$) along the mixing zone (**A**–**C**: ports L/D = 14, 15, and 16) for different kneading blocks: (**a**) KB45-3, (**b**) KB45-6, and (**c**) KB90-6 at various screw speeds (100 to 500 rpm).

The distributive mixing levels (in terms of the variance,$\;\sigma^{2}$) also depend on screw geometry, screw speed, and axial position. As foreseen, at each location we found that the higher the screw speed was, the higher the variance was; and as the average shear rate increases, so does the deformation of fluid elements. This effect strengthens as the flow progresses downstream, with an exception at L/D = 16, which unexpectedly shows an almost constant low variance. In order to understand the underlying reason for this behavior, data on normalized cross-polarized transmitted light intensity (calculated according to Equation (6)), which simulates the form birefringence, was plotted for all screw profiles and screw speeds at each port (see Figure 10). 

A higher level of form birefringence is observed at L/D = 14 and L/D = 15 for all screw profiles, indicating the presence of a dispersed rod-type morphology, while at L/D = 16 this effect is greatly reduced. This probably means that as the flow progresses along the kneading block, continuing melt stretching takes place and, eventually, Rayleigh disturbances develop followed by rupture of the rod-type particles and the creation of numerous spherical particles. Not only do the latter not create form birefringence, but if they become sufficiently small (in the sub-micron range), their contribution to turbidity is much reduced. In turn, this flattens the RTD curves and, consequently, affects the variance. Therefore, although the mixing indices proposed here work well, they are influenced by the size (specifically, the scattering cross-section) of the particles generated during flow along the screw.

**Figure 11:**
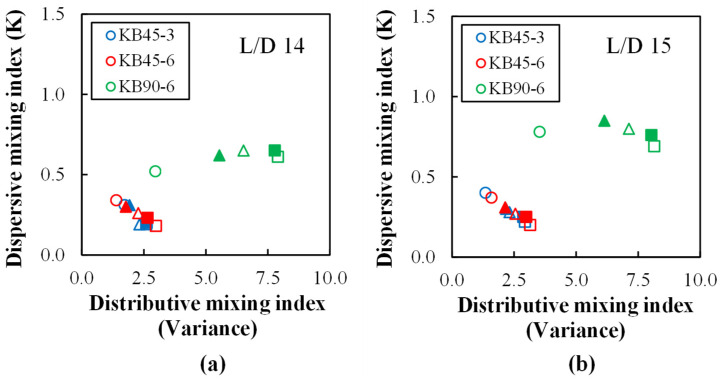
Mapping the mixing performance: dispersive mixing index (K) versus distributive mixing index ($\sigma^{2}$) for different kneading blocks (KB45-3, KB45-6, and KB90-6) and screw speeds (○—100 rpm, ▲—200 rpm, ∆—300 rpm, ■—400 rpm, and □—500 rpm) at ports: (**a**) L/D = 14 and (**b**) L/D = 15.

Figure 11 maps the dispersive and distributive mixing performance of the three kneading blocks investigated in this study, considering the distinct axial locations (except at L/D = 16 for the reasons discussed above) and the range of screw speeds tested. The data shift to higher levels of distributive and dispersive mixing as the melt progresses downstream. Generally, higher distributive and dispersive mixing levels are attained when using KB90-6, while the performance of KB45-3 and KB45-6 is virtually undistinguishable, i.e., the thickness of the KB45 disks (either 3 or 6 mm) does not seem to impart a significant effect. The highest dispersive mixing levels are attained with lower speeds due to the associated higher residence times, whereas distributive mixing is promoted by higher speeds.

## 5. Conclusions

In this work, an on-line optical detection system followed in real time the melt mixing behavior of a diluted polymer blend along a kneading section of a co-rotating twin screw extruder. The system relies on the light scattering and retardation produced by the particles of the dispersed phase, yielding information on the number (as turbidity) and shape (as form birefringence) of particles. Inserting the second phase component as a pulse at the entrance and monitoring its exit at various axial locations along the mixing zone, residence time distribution curves were obtained. The parameter K (a constant in the pulse curve related to the area under an RTD curve) and the variance of the RTD curves were used as dispersive and distributive mixing indices, respectively.

Data concerning the (equivalent to) melt pressure, melt temperature, specific mechanical energy, and minimum and mean residence time along each kneading zone revealed the magnitude of the effects of changing screw speed and kneading block geometry on the flow characteristics which, in turn, should reflect on the extent of the mixing developed.

The parameter K and the variance of the RTD curves showed that higher dispersive mixing levels were attained for the mixing zone consisting of disks staggered at 90°, as it is associated to higher residence times than those having lower angles (45°). Distributive mixing increases with increasing screw speed, although it was shown that the indices may be influenced by the size (specifically, the scattering cross-section) of the particles generated during flow along the screw. These results are well in-line with the observations reported in the literature, and thus validate the experimental approach proposed in this work.

Therefore, the set-up and methodology used here can contribute to quickly assess the mixing performance of screw zones, which is useful for assembling a suitable screw configuration, setting the processing conditions, or designing new screw elements for twin-screw extruders. With the appropriate adaptations, the set-up could also be used in batch mixers and other types of polymer processing equipment.

## Data Availability

Not applicable.
